# Sex-dependent effects of maternal corticosterone and SSRI treatment on hippocampal neurogenesis across development

**DOI:** 10.1186/s13293-017-0142-x

**Published:** 2017-06-02

**Authors:** Aarthi R. Gobinath, Joanna L. Workman, Carmen Chow, Stephanie E. Lieblich, Liisa A. M. Galea

**Affiliations:** 1 Program in Neuroscience, University of British Columbia, 2215 Wesbrook Mall, Vancouver, BC V6T 1Z3 Canada; 2 Department of Psychology, University of British Columbia, 2136 West Mall, Vancouver, BC V6T 1Z4 Canada; 3 Centre for Brain Health, University of British Columbia, 2215 Wesbrook Mall, Vancouver, BC V6T 1Z3 Canada; 4 Present Address: Department of Psychology, University at Albany, State University of New York, 1400 Washington Ave., Albany, NY 12222 USA

**Keywords:** Postpartum corticosterone, Fluoxetine, Doublecortin, Sex differences, Hippocampus, SSRIs, Neurogenesis, Dentate gyrus, Postpartum depression

## Abstract

**Background:**

Postpartum depression affects approximately 15% of mothers and represents a form of early life adversity for developing offspring. Postpartum depression can be treated with prescription antidepressants like fluoxetine (FLX). However, FLX can remain active in breast milk, raising concerns about the consequences of neonatal FLX exposure. The hippocampus is highly sensitive to developmental stress, and males and females respond differently to stress at many endpoints, including hippocampal plasticity. However, it is unclear how developmental exposure to FLX alters the trajectory of hippocampal development. The goal of this study was to examine the long-term effects of maternal postpartum corticosterone (CORT, a model of postpartum depression) and concurrent FLX on hippocampal neurogenesis in male and female offspring.

**Methods:**

Female Sprague-Dawley rat dams were treated daily with either CORT or oil and FLX or saline from postpartum days 2–23. Offspring were perfused on postnatal day 31 (pre-adolescent), postnatal day 42 (adolescent), and postnatal day 69 (adult). Tissue was processed for doublecortin (DCX), an endogenous marker of immature neurons, in the dorsal and ventral hippocampus.

**Results:**

Maternal postpartum CORT reduced density of DCX-expressing cells in the dorsal hippocampus of pre-adolescent males and increased it in adolescent males, suggesting that postpartum CORT exposure disrupted the typical progression of the density of DCX-expressing cells. Further, among offspring of oil-treated dams, pre-adolescent males had greater density of DCX-expressing cells than pre-adolescent females, and maternal postpartum CORT prevented this sex difference. In pre-adolescent females, maternal postpartum FLX decreased the density of DCX-expressing cells in the dorsal hippocampus compared to saline. As expected, maternal CORT reduced the density of DCX-expressing cells in adult female, but not male, offspring. The combination of maternal postpartum CORT/FLX diminished density of DCX-expressing cells in dorsal hippocampus regardless of sex or age.

**Conclusions:**

These findings reveal how modeling treatment of postpartum depression with FLX alters hippocampal neurogenesis in developing offspring differently depending on sex, predominantly in the dorsal dentate gyrus and earlier in life.

## Background

Early life adversity can impose detrimental effects on the neurobiological outcome of the developing child. One of the most potent mediators of early health is quality of maternal care. Maternal mood disorders, such as postpartum depression (PPD), can disrupt the mother–infant bond as well as negatively affect maternal caregiving behaviors (reviewed in [1]). For this reason, PPD may be considered a form of early life adversity. The adverse effects of untreated PPD include increased risk for depression among adolescent boys and girls ([2]—proportion of children of mothers with depression [3]— in comparison to children of mothers with no history of depression), propensity for violent behavior ([4] —in comparison to children of mothers without depression), and lower IQ scores ([5] —meta-analysis). However, treating PPD is complicated by the poorly understood consequences of neonatal exposure to prescribed antidepressants such as selective serotonin reuptake inhibitors (SSRIs). In some studies, there are either minimal or positive effects of maternal SSRI exposure observed in children. For example, neonatal SSRI exposure had minimal impact on body weight [6] and enhanced language development in comparison to no exposure to SSRIs [7]. Additionally, children with neonatal SSRI exposure exhibited fewer behavior problems (hyperactivity and inattention) than children of mothers with untreated depression [8]. However, maternal SSRI use has been linked to delayed psychomotor development in infants [9], increased internalizing behavior in children (i.e., behaviors predisposing anxiety and depression; [10]), and a higher risk for autism spectrum disorders in children [11]. These findings may be consistent with preclinical findings from our laboratory that maternal postpartum fluoxetine (FLX) exposure increased anxiety-like behavior in young adult male offspring [12]. However, as in the clinical literature, preclinical research has yielded mixed results regarding maternal SSRI exposure on offspring outcome depending on whether a model of concurrent maternal depression was used, sex of the offspring studied, and age examined (reviewed in [13]). To this end, this study aims to contribute a better understanding of how maternal SSRI exposure in a rat model of PPD affects male and female development (specifically hippocampal neurogenesis) at three different ages.

The hippocampus is highly sensitive to the effects of stress and stress hormones (i.e., glucocorticoids) throughout the lifespan, including during early development (reviewed in [14]). After parturition, postnatal hippocampal neurogenesis (from birth until weaning; approximately 21 days) is necessary to develop the hippocampus into its fully matured form. In fact, 85% of granule cells forming the dentate gyrus are born during this postnatal period [15]. After this developmental period, the structural matrix of the dentate gyrus is formed, but the subgranular zone of the dentate gyrus continues to generate new neurons throughout the lifespan. Different forms of early life adversity can affect neurogenesis in the dentate gyrus during the postnatal period (maternal deprivation; [16]), adolescence (prenatal stress; [17]), and adulthood (maternal deprivation; [18, 19]). Doublecortin (DCX) is an endogenous, microtubule-associated protein important for neuronal migration [23] that is most highly expressed early in development and progressively tapers off throughout development and into adulthood. Early life adversity can disrupt this pattern of expression across the lifespan.

Given the well-established sex differences in stress responses (reviewed in [22]), it is perhaps not surprising that sex is an important factor in mediating the effects of early life adversity on hippocampal neurogenesis. For example, an acute bout of maternal deprivation (24 h on postnatal day 3) increased the density of DCX-expressing cells in pre-adolescent male rats but decreased it in pre-adolescent female rats [16]. However, by adulthood, this paradigm of maternal deprivation decreased the density of immature neurons in male rats [18] but had no significant effect in females, suggesting that density of DCX-expressing cells had recovered in females [19]. However, longer periods of maternal separation (3 h/day) for the first two postnatal weeks transiently reduce DCX-positive cells at the end of maternal separation in male rat pups [20]. These findings indicate that early adversity via maternal deprivation had age- and sex-specific influences on neurogenesis throughout development, particularly in males. Notably, early life adversity does not always lead to a suppressive effect on neurogenesis but rather can alter the time course of neurogenesis over the lifespan in dynamic ways. However, a singular bout of maternal deprivation more closely models acute and severe neglect and does not model the voluntary and diminished quality of maternal care observed with PPD. PPD is associated with prolonged periods of disengaged maternal care, which includes neutral affect and withdrawal from the infant and sometimes negative maternal care, which includes negative affect and hostility directed to the infant (reviewed in [21]). The maternal neglect modeled by maternal separation/deprivation [16, 19, 20] therefore does not approximate the prolonged diminished quality of maternal care that occurs in PPD but rather, represents abrupt cessation of maternal care. Thus, one of the goals of the present study is to examine how postnatal adversity relevant to PPD (i.e., sustained reductions in maternal care and higher maternal glucocorticoid levels) affects hippocampal neurogenesis from post-weaning to adult stages of development in both sexes.

To investigate the effects of PPD on offspring development and neurogenesis, we used a rodent model of PPD. In this model, dams are treated daily with high levels of corticosterone (CORT) to induce a PPD-like phenotype, thus capitalizing on the well-established relationship between glucocorticoids and depression, including perinatal depression (reviewed in [14]). Treating postpartum rats with CORT consistently increases time spent away from the nest and reduces time spent nursing without completely absolutely depriving the pups of any maternal care [23–25]. Although other models of depression exist in the literature, such as chronic unpredictable stress, these paradigms applied in the postpartum would either force the dam to be away from her pups (making it difficult to distinguish the effects of maternal separation from maternal “depression”). Or, if pups were kept with the dam during stress exposure, this would directly inflict stress on the pups themselves (making it difficult to distinguish the effects of direct postnatal stress from maternal “depression”). Unlike these models, the CORT-induced model of PPD induces depressive-like behavior with minimal separation of the dams from the offspring (<1 min to perform injections) and results in the dam voluntarily withdrawing from her offspring as well as higher levels of CORT in the milk [25, 38], which mimic key features of PPD in women (reviewed in [21]). When examining the offspring outcome, maternal postpartum CORT decreased hippocampal cell proliferation in males, but not females, just after weaning [24]. By adolescence, however, maternal postpartum CORT did not significantly affect survival of new cells produced at weaning in either sex [24]. However, whether these early reductions in cell proliferation perturbed neurogenesis (differentiation into new neurons) at the time of weaning or later in life were not determined. The present study aims to address this gap by examining how maternal CORT exposure affects density of immature neurons in the hippocampus after weaning as well as in adolescence and adulthood in both sexes.

In contrast to the body of research examining developmental stress and its effects on the hippocampus, there is limited research evaluating how developmental FLX exposure affects the hippocampus. Treating dams with FLX in the postpartum reversed the suppressive effect of prenatal stress on hippocampal DCX-expressing cells in both adolescent male and female offspring [17]. However, this interaction between prenatal stress and postpartum FLX exposure did not persist to adulthood as maternal postpartum FLX decreased number of DCX-expressing cells in offspring also exposed to prenatal stress, particularly in the males [29]. We recently reported that FLX given to the dam during the postpartum period increased density of DCX-expressing cells in the dorsal hippocampus of adult male offspring but not in adult female offspring [12]. It remains unclear, however, whether maternal postpartum FLX, either alone or in combination with CORT alters hippocampal neurogenesis across development in a rodent model relevant to PPD. To investigate this, we administered high CORT to dams to induce a depression-like phenotype [24, 25, 27, 28] as well as FLX to model antidepressant treatment of postpartum CORT in dams [28]. While there has been considerable investigation of how stress affects hippocampal neurogenesis in the early developmental periods (prenatal and postnatal) as well as in later ages (adulthood and aging), relatively little is known about how hippocampal neurogenesis proceeds through pre-adolescent and adolescent time periods. Moreover, how developmental exposure to CORT and/or FLX affects the neurogenic trajectory in the hippocampus is also poorly understood. Thus, we investigated male and female offspring after weaning (pre-adolescence), in adolescence, and in adulthood. We hypothesized maternal postpartum CORT and FLX would alter the time course of hippocampal neurogenesis throughout development differently in males and females, and that both treatments could potentially interact such that FLX may prevent disruptions that arise following maternal postpartum CORT exposure. More specifically, we predicted that males would be more sensitive to CORT and FLX than females, given the established literature demonstrating that males are more vulnerable to early life adversity in a variety of endpoints (reviewed in [14]).

## Methods

### Animals

Thirty-two adult female Sprague-Dawley rats (2–3 months old) and 16 adult male Sprague-Dawley rats (2–3 months old, Charles River) were initially housed in same-sex pairs with aspen chip bedding in the Centre for Disease Modeling at University of British Columbia. Rats were maintained in a 12:12 h light/dark cycle (lights on at 7:00 am) and given rat chow (Jamieson’s Pet Food Distributors Ltd, Delta, BC, Canada) and tap water ad libitum. All protocols were in accordance with ethical guidelines set by Canada Council for Animal Care and were approved by the University of British Columbia Animal Care Committee.

### Breeding procedures

For breeding, two females and one male were paired daily between 5:00 and 7:00 pm. Females were vaginally lavaged each morning between 7:30 and 9:30 am. Upon identification of sperm in the lavage sample, females were considered pregnant, weighed, and single housed into clean cages with autoclaved paper towels and an enrichment tube.

### Maternal treatments

1 day after birth (birth day = postnatal day 0), all litters were culled to 5 males and 5 females. If there were not enough males or females in one litter, pups were cross-fostered from a dam that gave birth the same day. If there were not enough pups available to support a five male and five female litters, then dams maintained a sex-skewed or smaller litters (this happened twice with both being in the CORT/saline group). Dams were randomly assigned to one of four treatment groups: (1) oil/saline; (2) oil/FLX; (3) CORT/saline; (4) CORT/FLX. Beginning on postpartum day 2, dams received daily injections of either subcutaneous CORT (40 mg/kg) or sesame oil (1 ml/kg). Dams also received a second injection of either intraperitoneal FLX (10 mg/kg) or saline (1 ml/kg). Injections occurred daily for 22 consecutive days. The effects of maternal postpartum CORT/saline on depressive-like behavior and maternal behavior were verified in the dam, and data about the effects of maternal postpartum CORT and FLX on serum CORT levels in dams as well as body mass of dams and pre-weaning offspring have been published separately [28]. Dams received both injections in succession between 11am and 2 pm.

### Drug preparation

An emulsion of CORT (Sigma-Aldrich, St. Louis, MO, USA) was prepared every 2–3 days by mixing CORT with ethanol and then adjusting with sesame oil to yield a final concentration of 40 mg/ml of CORT in oil with 10% ethanol. The dose was chosen because it reliably induces a depressive-like phenotype in dams, disrupts maternal care, and affects offspring development [23, 24]. To control for CORT, vehicle injections consisted of 10% ethanol in sesame oil (referred to as “oil”). FLX (Sequoia Research Products, Pangbourne, UK) was prepared every 2–3 days by dissolving in dimethyl sulfoxide (DMSO; Sigma Aldrich) and adjusted with 0.9% saline to yield a final concentration of 10 mg/ml FLX in saline with 10% DMSO. This dose of FLX was chosen based on work illustrating that this dose increased brain derived neurotrophic factor and cell proliferation in the hippocampus and amygdala after 21 days of injections in both male and female rodents [30]. To control for FLX, vehicle injections consisted of 10% DMSO in 0.9% saline.

### Offspring tissue collection

For the pre-adolescent offspring, pups remained group-housed with litter mates until perfusion. For the adolescent and adult offspring, pups were weaned on postnatal day 24 and pair-housed with an unrelated, same-sex cage mate whose mother received the same treatment. Besides weekly cage changing, offspring remained undisturbed until perfusion. Based on the four maternal treatments described above, rats were used from each of the following groups were utilized at pre-adolescence, adolescence, and adult time points (*n* = 124): male oil/saline offspring, *n* = 5–7; male oil/FLX offspring, *n* = 5; male CORT/saline offspring, n = 5; male CORT/FLX offspring, *n* = 5; female oil/saline offspring, *n* = 5; female oil/FLX offspring, *n* = 5; female CORT/saline offspring, *n* = 5; female CORT/FLX offspring, n = 5.

**Fig. 1:**
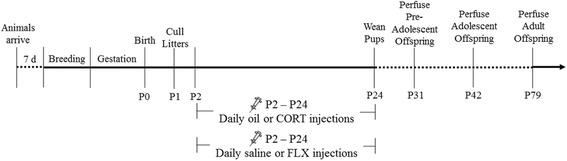
Timeline of experiment. (not to scale)

Pre-adolescent offspring were perfused on postnatal day 31. Adolescent offspring were perfused on postnatal day 42 because this time point is considered to be mid-adolescence [31–33]. Adult offspring were perfused on postnatal day 69. On the day of perfusion, rats were weighed and then given an overdose of Euthanyl (sodium pentobarbital). Rats were perfused with 60 ml cold 0.9% saline followed by 120 ml cold 4% paraformaldehyde. Brains were extracted and post-fixed using 4% paraformaldehyde overnight at 4 °C. Brains were then transferred to 30% sucrose in phosphate buffer at 4 °C until they sank to the bottom. Brains were rapidly frozen with dry ice, were sectioned using a freezing microtome (Leica, Richmond Hill, ON, Canada) at 40 μm, and were collected in series of 10. Sections were stored in antifreeze (ethylene glycol/glycerol; Sigma) and were stored at −20 °C until processing. For an overview of experimental procedures, refer to Fig. 1.

### DCX immunohistochemistry

Sections were rinsed 5 × 10 min in 0.1 M phosphate-buffered saline (PBS), were treated with 0.3% hydrogen peroxide in dH_2_O for 30 min, and were incubated at 4 °C in primary antibody solution: 1:1000, goat anti-DCX (Santa Cruz Biotechnology, Santa Cruz, CA, USA) with 0.04% Triton-X in PBS, and 3% normal rabbit serum for 24 h. Sections were then rinsed 5 × 10 min in 0.1 M PBS and were transferred to a secondary antibody solution with 1:500, rabbit anti-goat (Vector Laboratories, Burlington, ON, Canada) in 0.1 M PBS for 24 h at 4 °C. Then, sections were washed 5 × 10 min in 0.1 M PBS and were incubated in ABC complex (ABC Elite Kit; 1:1000; Vector) for 4 h. Sections were then washed in 0.175 M sodium acetate buffer 2 × 2 min. Finally, sections were developed using diaminobenzidine in the presence of nickel (DAB Peroxidase Substrate Kit, Vector), mounted on slides, and dried. Sections were then dehydrated and coverslipped with Permount (Fisher Scientific).

DCX-expressing cells were quantified in 3 dorsal sections (−2.76 to −4.68 mm below bregma) and 3 ventral sections (−5.52 to −6.60 mm below bregma) using the ×40 objective using an Olympus CX22LED brightfield microscope. Areas of these sections were quantified using ImageJ (NIH, Bethesda, MD, USA) and were used for density calculations (number of cells per mm^2^).

### Data analyses

Data were analyzed using repeated measures ANOVA with hippocampal region (dorsal, ventral) as the within-subject factor and age, sex, maternal postpartum CORT, and maternal postpartum FLX as between-subject factors. Newman-Keuls tests were conducted for post-hoc comparisons which controls for multiple pair-wise comparisons in a step-wise fashion. Because we had hypotheses that sex, CORT, and FLX would interact, a priori comparisons were conducted and subjected to Bonferroni corrections. All data were analyzed using Statistica software (v. 9, StatSoft, Inc., Tulsa, OK, USA). All effects were considered statistically significant if *p* ≤ 0.05.

## Results and discussion

Our statistical analysis was a comprehensive five-way ANOVA which generated 31 possible main effects and interactions. Of these possibilities, ten main and interacting effects were statistically significant, with three main effects (age, sex, and region), four two-way interactions (age × sex, age × CORT, region × age, area × CORT), two three-way interactions (region × CORT × FLX, and region × age × CORT), and finally the four-way interaction (region × age × sex × CORT). There was also a trend for a four-way interaction (region × age × sex × FLX) and limited comparisons were conducted. These significant interactions are summarized in Table 1.

**Table 1:** Summary of statistical interactions

Significant effects from omnibus ANOVA			*p* value
Interaction between region, age, sex, and maternal postpartum CORT			
Dorsal hippocampus	Pre-adolescent	♂: *oil > CORT*	*p < 0.001*
		♀: oil vs. CORT	n.s.
	Adolescent	**♂**: *oil < CORT*	*p* *= 0.03*
		**♀**: *oil < CORT*	*p = 0.02*
	Adult	♂: oil vs. CORT	n.s.
		♀: *oil > CORT*	*p = 0.02*
Ventral hippocampus	Pre-adolescent	♂: oil vs. CORT	n.s.
		♀: oil vs. CORT	n.s.
	Adolescent	♂: *oil < CORT*	*p = 0.003*
		♀: oil vs. CORT	n.s.
	Adult	♂: oil vs. CORT	n.s.
		♀: oil vs. CORT	n.s.
Interaction between region, age, sex, and maternal postpartum FLX			
Dorsal hippocampus	Pre-adolescent	♂: saline vs. FLX	n.s.
		♀: *saline < FLX*	*p = 0.003*
Interaction between region, maternal postpartum CORT, and FLX			
Dorsal hippocampus	*CORT/saline > CORT/FLX*		*p = 0.035*
	*Oil/FLX > CORT/FLX*		*p = 0.041*
Ventral hippocampus	*Oil/FLX < CORT/FLX*		*p = 0.032*

*CORT* corticosterone, *FLX* fluoxetine, *n.s.*, non-significant. Significant comparisons are in *italic*s

### Maternal postpartum CORT altered the density of DCX-expressing cells depending on age, sex, and hippocampal sub-region (interaction between region, age, sex, and maternal postpartum CORT)

**Fig. 2:**
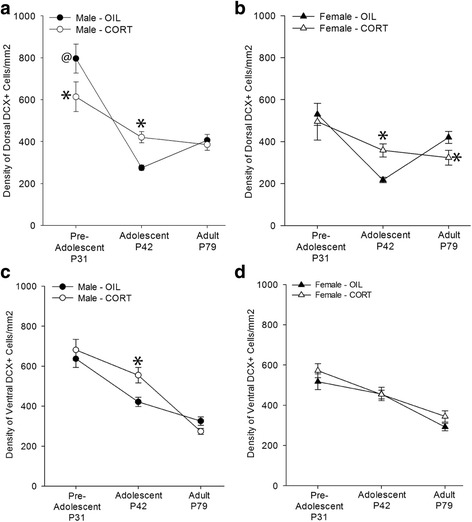
Mean + SEM density of DCX-expressing cells/mm^2^ in A–D. **a** In dorsal hippocampus, maternal postpartum CORT decreased the density of DCX-expressing cells in pre-adolescent males and increased it in adolescent males compared with oil. Males of oil-treated dams also had a greater density of DCX-expressing cells compared with females of oil-treated dams. **b** In the dorsal hippocampus of adolescent females, maternal postpartum CORT increased the density of DCX-expressing cells compared with oil. However, in adulthood, maternal postpartum CORT decreased the density of DCX-expressing cells in females. **c** In the ventral hippocampus of adolescent males, maternal postpartum CORT increased the density of DCX-expressing cells compared with oil. **d** In the ventral hippocampus of females, maternal postpartum CORT did not significantly alter the density of DCX-expressing cells. @*p* < 0.05, males vs. females; *p < 0.05, oil vs. CORT; *n* = 5–7/sex/group

Maternal postpartum CORT decreased the density of DCX-expressing cells in the dorsal hippocampus of pre-adolescent males in comparison to oil [*p* < 0.001, Cohen’s *d* = 0.83; area, age, sex, and maternal postpartum CORT interaction: F (2, 98) = 4.59, *p* = 0.01; Fig. 2a). Among oil-exposed offspring, pre-adolescent males had a greater density of DCX-expressing cells in comparison to pre-adolescent females in dorsal hippocampus (*p* < 0.001, Cohen’s *d* = 1.37). Maternal postpartum CORT exposure prevented this sex difference in pre-adolescent offspring (*p* = 0.06; Fig. 2a, b). Further, maternal postpartum CORT increased the density of DCX-expressing cells in the dorsal hippocampus of both adolescent male and female offspring compared with oil (males: *p* = 0.003, Cohen’s *d* = 2.18; females: *p* = 0.023, Cohen’s *d* = 1.98; Fig. 2a, b). In our prior study [12] we observed that maternal postpartum CORT, regardless of FLX, decreased density of dorsal DCX-expressing cells in female offspring, and an a priori comparison revealed the same result in this dataset (*p* = 0.019, Cohen’s *d* = 0.91). Maternal postpartum CORT significantly increased the density of DCX-expressing cells in the ventral hippocampus of adolescent males compared with oil (*p* = 0.003, Cohen’s *d* = 1.35; Fig. 2c) but not in females (*p* = 0.95; Fig. 2d).

### Density of DCX-expressing cells declined from pre-adolescence to adolescence except in the ventral hippocampus of CORT-exposed females (interaction between region, age, sex, and maternal postpartum CORT)

The density of DCX-expressing cells in the dorsal hippocampus decreased from pre-adolescence to adolescence regardless of sex or CORT exposure (*p*’s < 0.04, Cohen’s *d* = 0.66–3.31; Fig. 2a, b). In the ventral hippocampus of males, the density of DCX-expressing cells decreased from pre-adolescence to adolescence in oil- and CORT-exposed rats (*p*’s < 0.04, Cohen’s *d* = 0.86–1.97; Fig. 2c). In females, the density of DCX-expressing cells in the ventral hippocampus did not change significantly from pre-adolescence to adolescence in oil- or CORT-exposed rats (*p*’s > 0.08; Fig. 2d). There were no other significant main effects or interactions.

### Maternal postpartum CORT prevented the increase in density of DCX-expressing cells from adolescence to adulthood in the dorsal hippocampus (interaction between region, age, sex, and maternal postpartum CORT)

The density of DCX-expressing cells in the dorsal hippocampus increased from adolescence to adulthood in oil-exposed males (*p* = 0.046, Cohen’s *d* = 1.75) and females (*p* < 0.001, Cohen’s *d* = 2.71). This age-related increase, however, was not present in CORT-treated males (*p* = 0.92) or females (*p* = 0.84). The density of DCX-expressing cells in the ventral hippocampus decreased from adolescence to adulthood in CORT-exposed males (*p* < 0.001, Cohen’s *d* = 3.07), but not oil-exposed males (*p* = 0.26). The density of DCX-expressing cells in the ventral hippocampus decreased from adolescence to adulthood in oil-exposed females (*p* = 0.009; Cohen’s *d* = 1.95), but not CORT-exposed females (*p* = 0.17).

### Maternal postpartum FLX decreased the density of DCX-expressing cells in dorsal hippocampus of pre-adolescent female but not male offspring (interaction between region, age, sex, and maternal postpartum FLX)

**Fig. 3:**
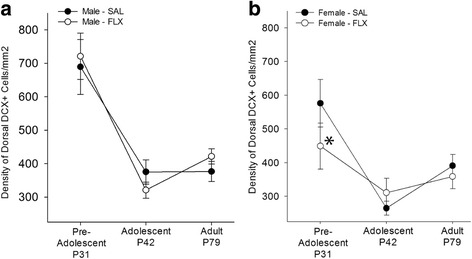
Mean + SEM density of DCX-expressing cells/mm^2^ in **a**, **b. a** Maternal postpartum FLX did not significantly alter the density of DCX-expressing cells in male offspring. **b** In pre-adolescent female offspring, maternal postpartum FLX decreased the density of DCX-expressing cells in the dorsal hippocampus compared with SAL. There were no other significant effects of sex, CORT, or FLX in ventral hippocampus. *p < 0.05, saline vs. FLX; *n* = 5–7/sex/group

A priori comparisons revealed that maternal postpartum FLX decreased the density of DCX-expressing cells in comparison to saline in the dorsal hippocampus of pre-adolescent females (*p* = 0.003, Cohen’s *d* = 0.58; Fig. 3b) but not males [*p* = 0.45; area, age, sex, and maternal postpartum FLX interaction: F (2, 98) = 2.74; *p* = 0.069; Fig. 3a]. Additionally, among offspring exposed to maternal postpartum saline, pre-adolescent males had a greater density of DCX-expressing cells than pre-adolescent females in the dorsal hippocampus (*p* = 0.008, Cohen’s *d* = 0.47).

### Maternal postpartum FLX prevented the increase in density of DCX-expressing cells from adolescence to adulthood in dorsal hippocampus of females (interaction between region, age, sex, and maternal postpartum FLX)

In the dorsal hippocampus, density of DCX-expressing cells increased from adolescence to adulthood among saline-exposed females (*p* = 0.003, Cohen’s *d* = 1.36) but not FLX-exposed females (*p* = 0.273). In the ventral hippocampus, density of DCX-expressing cells decreased similarly in offspring regardless of sex or FLX exposure (*p*’s <0.01, Cohen’s *d* = 1.23–2.48). No other comparisons were statistically significant after performing Bonferroni corrections.

### Maternal postpartum CORT and FLX decreased the density of DCX-expressing cells in dorsal hippocampus, but increased it in ventral hippocampus (interaction between region, CORT, and FLX)

**Fig. 4:**
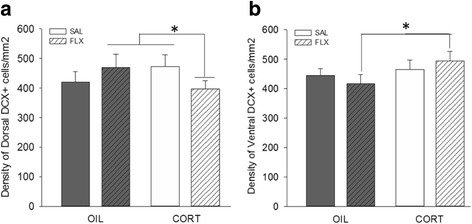
Mean + SEM density of DCX-expressing cells/mm^2^ in **a**, **b. a** Maternal postpartum CORT and FLX decreased the density of DCX-expressing cells in the dorsal hippocampus compared with CORT only and FLX only. **b** In the ventral hippocampus, maternal postpartum CORT and FLX increased the density of DCX-expressing cells in comparison to maternal postpartum FLX only. *p < 0.05. CORT/FLX vs. CORT only or FLX only; *n* = 5–7/sex/group

Regardless of age and sex, maternal postpartum CORT and FLX decreased the density of DCX-expressing cells in the dorsal hippocampus compared with CORT alone (*p* = 0.035, Cohen’s *d* = 0.41) and with FLX alone [*p* = 0.041, Cohen’s *d* = 0.36; area, CORT, and FLX interaction: F (1, 98) = 13.787, *p* = 0.003; Fig. 4a]. In the ventral hippocampus, the combination of maternal postpartum CORT and FLX increased the density of DCX-expressing cells compared to FLX exposure alone (*p* = 0.032, Cohen’s *d* = 0.44; Fig. 4b).

### Males weighed more than females in adolescence and adulthood

Male offspring weighed more than female offspring in adolescence (*p* < 0.001, Cohen’s *d* = 2.03) and adulthood (*p* < 0.001, Cohen’s *d* = 5.58) but not as pre-adolescents (*p* = 0.532; interaction between age and sex: F (2, 85) = 105.78; *p* < 0.001; Table 2). There were no other significant main effects of or interactions between age, sex, maternal postpartum CORT, or maternal postpartum FLX (all *p*’s > 0.21).

**Table 2:** Body mass (g) ± SEM. Males had a greater body mass than females in both adolescence and adulthood but not as pre-adolescents

	Pre-adolescent	Adolescent	Adult
Male–oil/SAL	122.2 ± 0.8	250.0 ± 7.3*	524.7 ± 12.6^@^
Male–oil/FLX	130.7 ± 9.0	248.2 ± 8.0*	529.4 ± 20.3^@^
Male–CORT/SAL	118.7 ± 10.4	236.0 ± 5.9*	543.3 ± 24.8^@^
Male–CORT/FLX	112.6 ± 9.2	234.6 ± 17.5*	553.0 ± 38.3^@^
Female–oil/SAL	118.4 ± 1.5	195.8 ± 5.3	307.7 ± 10.2
Female–oil/FLX	120.0 ± 2.6	159.0 ± 24.1	300.0 ± 10.8
Female–CORT/SAL	111.0 ± 6.1	186.4 ± 10.1	327.0 ± 16.0
Female–CORT/FLX	106.0 ± 10.2	190.4 ± 5.5	308.0 ± 13.7

*p < 0.05, adolescent males vs. adolescent females; ^@^ *p* < 0.05, adult males vs. adult females

## Discussion

Here, we show that maternal postpartum CORT and FLX affected density of DCX-expressing cells in males and females differently from pre-adolescence to early adulthood and that the dorsal hippocampus was more sensitive than the ventral hippocampus to these maternal treatments. Consistent with our hypothesis, maternal postpartum CORT predominantly affected males early in life such that it reduced the density of dorsal DCX-expressing cells in pre-adolescent males and increased density of dorsal DCX-expressing cells in adolescent males as well as females. On the other hand, maternal postpartum FLX predominantly affected females earlier in life as it decreased density of DCX-expressing cells in pre-adolescent females in the dorsal hippocampus and attenuated the age-related increase in density of DCX-expressing cells from adolescence to adulthood. Regardless of age and sex, maternal postpartum CORT and FLX decreased density of DCX-expressing cells in dorsal hippocampus and increased density of DCX-expressing cells in ventral hippocampus. Furthermore, maternal postpartum CORT reduced the density of DCX-expressing cells in adult females, consistent with previous findings [12]. Collectively, these findings reveal that maternal postpartum CORT and FLX can impact density of DCX-expressing cells earlier in the lifespan in males and females whereas the combination of CORT and FLX may have more general effects on density of DCX-expressing cells regardless of age and sex.

### Maternal postpartum CORT decreased density of DCX-expressing cells in pre-adolescent males and adult females but increased density of DCX-expressing cells in adolescent offspring

Maternal postpartum CORT decreased density of DCX-expressing cells in the dorsal hippocampus of pre-adolescent male but not female offspring. By adolescence, maternal postpartum CORT generally enhanced density of DCX-expressing cells in both sexes (sub-region differences are further discussed below). However, by adulthood, the effect of maternal postpartum CORT was no longer apparent in males but it reduced density of DCX-expressing cells in females. This extends previous work with this model which found that maternal postpartum CORT diminished hippocampal cell proliferation only in pre-adolescent males but not in females whereas maternal postpartum CORT did not affect survival of these cells produced post-weaning [24]. The detrimental effect of maternal postpartum CORT on density of DCX-expressing cells in males is in line with broad findings that males are more susceptible to adverse outcomes after perinatal complications and to neurodevelopmental disorders such as autism spectrum disorder (reviewed in [14, 34, 35]). Further, this CORT-induced reduction in neurogenesis is in line with findings that postnatal stress is associated with reductions in hippocampal neurogenesis and plasticity [18, 19, 36]. This reduction in neurogenesis may be related to disruption of the stress hyporesponsive period, an important period in postnatal development characterized by low levels of endogenous glucocorticoid levels which promotes optimal neural development [37]. Maternal postpartum CORT treatment can disrupt this period of quiescent glucocorticoid activity either indirectly via decreased maternal care [23, 28] or directly via increased CORT levels in the brain, serum, and stomach milk of the offspring [38]. Either mechanism could reduce neurogenesis after weaning. However, little is known about sex differences in the stress hyporesponsive period. There is some evidence that adrenocorticotropic hormone is capable of eliciting CORT secretion in female pups but not male pups during the stress hyporesponsive period [39, 40]. Additionally, female rat pups (20 days old) have higher basal CORT levels but blunted cold stress-induced CORT levels than males [41]. However, it is not clear how these sex differences in ontogeny of the HPA axis are related to hippocampal neurogenesis. It should be noted that although pre-adolescent females did not exhibit any changes in density of DCX-expressing cells, it remains possible that other measures of neurogenesis and/or plasticity were altered after maternal postpartum CORT exposure.

Interestingly, we found that maternal postpartum CORT increased density of DCX-expressing cells in adolescent offspring. In general, chronic stress or glucocorticoid exposure reduces hippocampal neurogenesis [42], but most of these studies have been conducted in rodents shortly after birth or in adulthood. Relative to either of these time points, there is a substantial gap in our understanding of neurogenesis in adolescence. Neurogenesis reaches peak levels in the rat dentate gyrus on postnatal day 6 and declines with age [43]. However, the relationship between the cells produced post-weaning and the cells that continually regenerate later in life has yet to be fully elucidated. One possible explanation for the increase in adolescent density of DCX-expressing cells is that it is a compensatory mechanism for a loss in plasticity after maternal postpartum CORT exposure to ultimately normalize density of DCX-expressing cells by adulthood under cage control conditions. Alternatively, neurogenesis continues outside of the dentate gyrus during puberty in the prefrontal cortex, nucleus accumbens, anteroventral periventricular nucleus, medial amygdala, and sexually dimorphic nucleus [44, 45]. Thus, another possible explanation is that the effect of maternal postpartum CORT increasing density of DCX-expressing cells in adolescence is part of a system-wide change in neurogenesis that will ultimately contribute to altered brain development. Furthermore, synaptic pruning is a critical developmental process that occurs throughout the brain throughout development including adolescence. It is important to note that a reduction in synaptic plasticity is a normal and necessary component to brain maturation. Thus, we caution against interpreting that the enhancing effect of maternal postpartum CORT on offspring density of DCX-expressing cells is favorable or beneficial for brain development because it is possible that maternal postpartum CORT disturbed this important culling process and resulted in aberrant density of DCX-expressing cells. Or, as discussed above, increased density of DCX-expressing cells could be a compensatory mechanism to promote density of DCX-expressing cells during adolescence after it was reduced in pre-adolescence. Alternatively, the opposing effects between pre-adolescence and adolescence may be influenced by the different housing conditions as pre-adolescent subjects were group-housed and adolescent subjects were pair-housed. There is some evidence that group housing versus isolation can influence neurogenesis in middle-aged rats [46] but not young adult male mice [47]. Nonetheless, housing differences between pre-adolescence and adolescence may be a contributing factor to the observed differences between these ages. Regardless of housing conditions, adolescence is a critical developmental period that deserves further research.

Maternal postpartum CORT decreased the density of DCX-expressing cells in dorsal hippocampus of adult females but did not affect density of DCX-expressing cells in adult males. This observation in adult females is consistent with our prior study [12] which examined adult male and female offspring and changes in neurogenesis after a battery of behavioral tests and exposure to dexamethasone. Thus, despite the repeated behavioral and endocrine challenges, present and prior studies indicate that maternal postpartum CORT reduced neurogenesis in adult females regardless of basal conditions (the present study) or challenging conditions (i.e., stress and/or novelty; [12]). Given the relationship between neurogenesis in the hippocampus and depression [48], the decreased neurogenesis in adult females exposed to maternal postpartum CORT may be related to the increased basal levels of serum CORT observed in adult female but not male offspring in this model [26]. Indeed, reductions in hippocampal neurogenesis have been mechanistically linked to abnormalities in the HPA axis at least in male mice [49]. This connection between endocrine and neural endophenotypes of depression may partly explain the propensity for depression in girls of mothers with depression [50]. Although the functional significance of reduced neurogenesis in dorsal hippocampus is not clear, this observation does indicate that female offspring are vulnerable to the effects of maternal postpartum CORT, and, unlike male offspring, manifest later in life. Collectively, these findings emphasize both pre-adolescence and adolescence as critical periods for maternal postpartum CORT effects on density of DCX-expressing cells in male offspring whereas the effects of maternal postpartum CORT on female neurogenesis emerge later in life during adolescence and adulthood.

### Maternal postpartum FLX decreased density of DCX-expressing cells in pre-adolescent females

Maternal postpartum FLX decreased density of DCX expressing cells in dorsal hippocampus of pre-adolescent females, although this finding had only a medium effect size. This is particularly interesting because our previous findings indicated that males were more vulnerable than females to the effects of maternal postpartum FLX treatment in terms of anxiety-like behavior and HPA axis dysregulation [12]. However, our present findings indicate that females are also vulnerable to the effects of maternal postpartum FLX early in life and that these effects manifest differently than in males. This female-specific vulnerability to maternal SSRI exposure early in development has also been observed in clinical research with maternal SSRI exposure reducing reelin expression in umbilical cord serum in female infants but not in male infants [51]. Based on the current state of research, the mechanism explaining early female vulnerability to maternal fluoxetine exposure is unclear. A lower dose of maternal postpartum FLX (5 mg/kg; s.c.) delays pubertal onset in female rats [55] and increases sexual behavior in adult female rats [57]. Thus, maternal postpartum FLX can impact development of female endocrine physiology which could alter hippocampal plasticity. It should be noted that different studies using a lower dose of FLX (5 mg/kg; s.c.) in dams found that maternal postpartum FLX has no effect on density of DCX-expressing cells in males or females at weaning [56], in adolescence [17], or in adulthood ([58]; only males examined). This suggests that only higher doses of FLX alter density of DCX-expressing cells in pre-adolescent offspring. Although the mechanism underlying how different doses of maternal postpartum FLX account for endocrine disturbances and hippocampal neurogenesis is not clear, maternal postpartum FLX can impact neurogenesis in pre-adolescent female offspring and highlights that both sexes need to be studied in this field of research.

### Maternal postpartum CORT and FLX together decreased density of DCX-expressing cells in dorsal but increased density of DCX-expressing cells in the ventral hippocampus in male and female offspring

Maternal postpartum FLX also potentiated the effects of maternal postpartum CORT, demonstrating that developmental FLX can interact with a model relevant for PPD to affect offspring density of DCX-expressing cells. This finding also underscores the importance of studying the effects of antidepressant exposure within a disease model. Maternal postpartum CORT and concurrent FLX increased density of DCX-expressing cells in the ventral hippocampus. This bolstering effect of maternal postpartum FLX is in line with numerous studies observing treatment of adult rats with FLX increases neurogenesis [59, 60]. Interestingly, maternal postpartum CORT and concurrent FLX also resulted in a decreased density of dorsal DCX-expressing cells. Although treating adult rodents with FLX typically increases hippocampal neurogenesis, the present study utilized a different route of FLX exposure (i.e., via the mother during postnatal development) which affects the brain differently than adult exposure.

This effect of maternal postpartum CORT and concurrent FLX reducing density of DCX-expressing cells in dorsal hippocampus is different from [12] which found that maternal postpartum CORT/FLX yielded opposing effects in the dorsal hippocampus of males and females undergoing behavioral testing. In our previous study [12], after a battery of behavioral tests and exposure to dexamethasone, maternal postpartum CORT and FLX increased dorsal density of DCX-expressing cells in adult males, and this increase was not observed in the present study. The difference in the methods and results between the present and prior studies indicate that neurogenesis varies from basal conditions (the present study) to challenging conditions (i.e., stress and/or novelty; [12]) particularly in males. DCX is expressed for up to 21 days after new neurons are produced [52], and the behavioral testing and dexamethasone exposure may have interacted with developmental exposure to CORT/FLX to affect the number of DCX-expressing cells. Furthermore, other studies demonstrate that experience, such as Morris water maze or radial arm maze training, alters measures of hippocampal plasticity that are not observed in cage controls ([53, 54], respectively). Collectively, this suggests that some of the effects of maternal postpartum CORT/FLX exposure on offspring hippocampal neurogenesis may not necessarily resolve by adulthood, but rather emerge after a challenge to the system (i.e., behavioral testing and/or dexamethasone exposure) as shown in our previous study. These findings indicate that concurrent maternal postpartum CORT and FLX will impact neurogenesis differently depending on experience with perhaps a greater impact in males than females. This set of observations has noteworthy functional implications regarding what maternal antidepressant use means within the context of maternal postpartum stress/depression. In considering use of rat models, it should be noted that the first 10 days of the postpartum period are equivalent to the third trimester [61, 62]. Nonetheless, it would be important for future studies to characterize the behavioral implications of maternal postpartum FLX exposure in both sexes at different time points.

### Dorsal versus ventral hippocampus: maternal postpartum CORT and/or FLX affect density of immature neurons respond differently depending on sub-region

In the present study, we found that neurogenesis in the dorsal hippocampus was more sensitive to maternal treatments with CORT or FLX than in the ventral hippocampus and that when concurrent of both drugs had opposing effects in dorsal and ventral hippocampus regardless of age or sex. The hippocampus is a heterogeneous structure with differences along the dorsal-ventral hippocampal axis in terms of function, gene expression, and neurotransmission (reviewed in [63]). The dorsal-ventral axis of the hippocampus is present even at day of birth [64] although its function is best understood in the adult brain. Generally, dorsal hippocampus is associated with learning and spatial navigation, and the ventral hippocampus is associated with stress regulation and affective behavior (reviewed in [63]). However, these functional roles are not exclusive in each pole of the hippocampus as dorsal hippocampus has been implicated in anxiety-behavior [65] and ventral hippocampus has been implicated in memory [66].

Interestingly, maternal postpartum CORT and FLX specifically (albeit independently) affected dorsal but not ventral hippocampus in the pre-adolescent offspring. In the adolescent offspring, maternal postpartum CORT affected the density of DCX-expressing cells in both dorsal and ventral hippocampus. However, in adult males, neither maternal postpartum CORT nor FLX alone significantly altered density of DCX-expressing cells in the dorsal or ventral hippocampus. Thus, one possibility is that the effects of maternal CORT and FLX treatment on ventral hippocampus were apparent under conditions when the HPA axis was in the process of maturing. Indeed, HPA axis reaches full maturity between P42 and P49 [33]. Thus, the effects in ventral hippocampus density of DCX-expressing cells alterations being present in adolescence may be reflective of maternal exposure to CORT and/or FLX interacting with the immature stress and gonadal hormone systems. As previously discussed, Gobinath et al. [12] found that maternal postpartum CORT alone and maternal postpartum FLX alone increased density of DCX-expressing cells, and this increase was selectively in the dorsal hippocampus of adult male rats. The functional implications of maternal postpartum CORT and FLX in pre-adolescent and adolescent offspring on behavior and neurogenesis are currently unknown, but these data indicate that outcomes during these earlier stages may be different than those seen in adulthood. Furthermore, our results indicate that the neurogenic potential of the dentate gyrus is altered with maternal postpartum exposure to CORT or FLX in both males and females at different stages in development, potentially influencing sex differences in risk for psychiatric disease at different ages.

## Conclusions

Collectively, these findings highlight that males and females are differentially vulnerable at different time periods during development to the effects of maternal postpartum CORT and/or maternal postpartum FLX. Maternal postpartum CORT decreased the density of DCX-expressing cells in pre-adolescent males but increased it in both adolescent males and females. Maternal postpartum FLX decreased the density of DCX-expressing cells in the dorsal hippocampus of pre-adolescent females but not males. As we have noted previously, maternal postpartum CORT decreased the density of DCX-expressing cells in the dorsal hippocampus of adult female but not male offspring. However, maternal postpartum FLX did not significantly affected adult offspring of either sex, suggesting that the effects present earlier in life were resolved by adulthood at least under cage control conditions. In contrast, the combination of maternal postpartum CORT and FLX together decreased the density of dorsal DCX-expressing cells and increased it in the ventral hippocampus regardless of age or sex. Thus, the combination of maternal postpartum CORT and FLX altered the density of DCX-expressing cells regardless of age and sex whereas each treatment independently impacted hippocampal density of DCX-expressing cells earlier in life depending on sex. These findings yield important implications for maternal antidepressant use in treating PPD and developmental outcome and highlight that early postnatal environments can differentially affect both sexes.

## Abbreviations

CORT: Corticosterone
DCX: Doublecortin
FLX: Fluoxetine
P: Postnatal day
PPD: Postpartum depression
SAL: Saline
